# Supplementary material for: The PI3K-AKT-VEGF Signaling Pathway Contributes to Doxorubicin-induced Chemotherapy-related Cognitive Impairment
Source: Curr Neuropharmacol. 2026 Apr 15;24(9):1331–46. doi: 10.2174/011570159X414493251121073748 (PMC13536702; doi:10.2174/011570159X414493251121073748)
Supplement: Supplementary file 1 [file CN-24-9-1331_SD1.pdf]

Current Neuropharmacology, 2026, *24*, 1331-1346

RESEARCH ARTICLE

The PI3K-AKT-VEGF Signaling Pathway Contributes to Doxorubicin-induced Chemotherapy-related Cognitive Impairment

Xue-chun Zhang[1,2,#], Ling-chen Jiang[1,2,#], Jun-xiao Song[1,2,#], Ji-chao Peng[3], Ke Ni[3], Fu-ming Shen[1,2,*] and Can-can Zhou[1,*]

[1]*Department of Pharmacy, Shanghai Tenth People's Hospital, Tongji University School of Medicine, Shanghai, 200040, China;* [2]*Department of Clinical Pharmacy, School of Basic Medicine and Clinical Pharmacy, China Pharmaceutical University, Nanjing, 211198, China;* [3]*School of Pharmacy, Shanghai University of Traditional Chinese Medicine, Shanghai, 201203, China*

© 2026 The Author(s). Published by Bentham Science Publisher. This is an open access article published under CC BY 4.0 https://creativecommons.org/licenses/by/4.0/legalcode

ARTICLE HISTORY

Received: June 02, 2025
Revised: October 11, 2025
Accepted: October 21, 2025

DOI:
10.2174/011570159X414493251121073748

Abstract: ***Introduction***: Chemotherapy-related cognitive impairment (CRCI) has been identified as one of the primary adverse effects of chemotherapy. Doxorubicin (DOX) is an anthracycline chemotherapeutic agent recognized as a fundamental component of chemotherapy. Nevertheless, limited research has been conducted to elucidate the neurotoxic mechanisms of DOX-mediated CRCI. We investigated the neurotoxic mechanisms of DOX by utilizing network toxicology and transcriptomic methods.

Methods: DOX-induced CRCI animal models were successfully established. Spatial learning and memory were assessed using the Morris water maze (MWM) test. The levels of neuronal and synapse-related proteins in the cortex and hippocampus were detected. To elucidate the molecular mechanisms underlying DOX-induced neurotoxicity, an integrated approach combining network toxicology with transcriptomic profiling was employed. Lastly, the neurotherapeutic potential of LY294002 in relieving DOX-induced BBB disruption, neuronal cell loss, and cognitive impairment was evaluated.

Results: Our findings demonstrate that DOX induces spatial learning and memory deficits and promotes neuronal cell loss, primarily by disrupting the blood-brain barrier (BBB) *via* the PI3K-AKT signaling pathway. Notably, we identified a significant upregulation of vascular endothelial growth factor (VEGF) within astrocytes in mice following DOX exposure.

Discussion: Pharmacological inhibition of PI3K with LY294002 significantly reduced VEGF expression, mitigated BBB disruption and neuronal loss, and consequently alleviated DOX-associated cognitive impairment.

Conclusion: DOX exerts neurotoxic effects by promoting VEGF oversecretion through the upregulation of the PI3K-AKT pathway. Inhibition of the PI3K-AKT pathway effectively mitigates these effects, thereby alleviating DOX-induced BBB disruption, neuronal cell loss, and cognitive impairment.

Keywords: Doxorubicin, chemotherapy-related cognitive impairment, blood-brain barrier, PI3K-AKT-VEGF pathway, astrocyte, neuronal cell loss.

1. INTRODUCTION

Chemotherapy-related cognitive impairment (CRCI), also termed "chemobrain," or “chemo fog”, is characterized by impairments in learning, attention, and working memory that occur during or following chemotherapy therapy [1]. Clinical data reveal that CRCI manifests across a range of cancer types, including leukemia, breast cancer, thyroid cancer, and prostate cancer [2-4]. For example, current studies report that over 75% of cancer survivors experience chemotherapy during active chemotherapy treatment. Furthermore, more than 35% of patients continue to suffer from CRCI after completing chemotherapy, with symptoms often persisting for months to years [5, 6]. The National Cancer Institute has identified CRCI as a remarkable side effect that significantly

*Address correspondence to these authors at the Department of Pharmacy, Shanghai Tenth People's Hospital, Tongji University School of Medicine, Shanghai, 200040, China. E-mails: zhoucan3420@tongji.edu.cn (C.c.Z.); fumingshen@tongji.edu.cn (F.m.S.)
#*These authors contributed equally to this work.*

Current Neuropharmacology

impedes survivors' return to normal life [7]. Doxorubicin (DOX), an anthracycline chemotherapeutic agent discovered in the 1950s, has emerged as a fundamental component of chemotherapy owing to its potent and broad anticancer activity [8]. However, its clinical application is heavily limited by severe side effects, including myelosuppression, neurotoxicity, cardiotoxicity, and hepatotoxicity. Previously, it was believed that DOX had limited ability to penetrate the blood-brain barrier and thus exerted indirect neurotoxicity by generating reactive oxygen species and producing inflammatory factors [9]. Interestingly, Licht *et al*. have reported that DOX can cross the blood-brain barrier via apical projections of neural stem cells [10]. Recent clinical and experimental studies have also demonstrated that DOX is detectable in the brain following peripheral administration [11, 12]. Previously, Manchon *et al.* demonstrated that DOX triggered direct neurotoxicity associated with the formation of DNA double-strand breaks and the downregulation of Breast Cancer Type 1 [13]. Park *et al.* reported that DOX-related cognitive impairment can also elevate mitochondrial ROS levels and induce calcium dysregulation [14]. However, the direct neurotoxic mechanisms of DOX-related cognitive impairment remain inadequately understood, and the potential mechanisms warrant further investigation.

Network toxicology is an emerging bioinformatics field that focuses on analyzing biological systems and multi-pathway regulatory mechanisms. RNA-sequencing (RNA-seq) analysis is one of the most widely used methods for estimating RNA expression changes in an organism under different conditions and for exploring key genes and potential candidate molecules. Currently, integrating network toxicology, RNA-seq analysis, and *in vivo* experiments is a promising strategy for elucidating potential toxicological mechanisms. For example, Xiao *et al.* combined network toxicology and transcriptomic analysis, revealing that vine tea-mediated hepatotoxicity was associated with oxidative stress and apoptosis [15]. Yang *et al.* used the same strategy and ascertained that benzo[a]pyrene triggered liver damage by disrupting the JAK2-STAT3 signaling pathway [16]. In the present work, we employed an integrative approach combining network toxicology, transcriptomic analysis, and *in vivo* experiments to investigate potential toxic mechanisms underlying DOX-induced CRCI. This study not only serves as a reference for understanding the toxicity of DOX but also identifies potential detoxification targets for DOX poisoning.

2. MATERIALS AND METHODS

2.1. Animals and Treatment

All experimental studies in mice were reviewed and approved by the Institutional Animal Care and Use Committee of Shanghai Tenth People's Hospital of Tongji University, Shanghai, China (Approval number: SHDSYY-2022-4835), and in accordance with the US National Research Council's "Guide for the Care and Use of Laboratory Animals". 8-10 weeks male C57BL/6J mice (22-25 g) were obtained from LINGCHANG BIOTECH (Shanghai, China) and maintained in cages with a 12/12-hour light/dark cycle at 22 ± 3°C. Water and food pellets are available *ad libitum*. The DOX-induced cognitive damage models were established as previously described [11]. The DOX dosage used in this study was calculated from its single maximum clinical dosage (60-75 mg/m^2) and was consistent with previous literature [17]. After one week of acclimatization, the mice were randomly divided into two groups. (1) The control group (Ctrl) received normal saline; (2) the DOX group received a single dose of DOX (25 mg/kg) via intraperitoneal injection (i.p.). After 72 hours, the MWM test was conducted, and the mice were sacrificed for sample collection. In the LY294002 experiment, the mice were randomly assigned to Ctrl, DOX, and DOX + LY294002 groups. Mice in the Ctrl group were intraperitoneally injected with normal saline; Mice in the DOX group were intraperitoneally injected with a single dose of DOX (25 mg/kg); Mice in the DOX + LY294002 group were treated with LY294002 (8 mg/kg/d) intraperitoneally before DOX exposure for 7 consecutive days. The dosage and treatment regimen of LY294002 were based on previous studies [18]. This study adheres to internationally accepted standards for animal research and follows the 3Rs principle. The ARRIVE guidelines were employed for reporting experiments involving live animals, promoting ethical research practices.

2.2. Morris Water Maze (MWM) Test

The Morris Water Maze test is a well-established method for assessing spatial learning and memory in rodents. In our study, we used a circular pool measuring 150 cm in diameter and 50 cm in depth. The pool was surrounded by white curtains, and distinct visual cues of various shapes were attached to the walls to help the mice orient themselves. Throughout the testing period, the ambient temperature was carefully maintained between 21-22°C. To make the water opaque and conceal the escape platform, we added titanium oxide (Macklin, T818992). During the pre-experimental phase, we placed a platform (9 cm wide) that protruded 1.5 cm above the water, making it easy to see. Starting positions and the platform location followed a specific layout. During the learning phase, the platform (9 cm in diameter) was submerged 1.5 cm below the water surface. Each mouse completed 4 trials each day with 30-minute breaks between trials. The hidden platform stayed in the same quadrant, but the start position varied each day according to a preset schedule. Each mouse had up to 60 seconds to find the hidden platform. If they failed to find the platform in time, we gently guided them to it and allowed them to stay there for 10 seconds. After a 5-day training period, a probe test is conducted on the sixth day. Here, we removed the platform and allowed the mice to swim freely for 60 seconds. The entire experiment was recorded using a video-tracking system (Shanghai Jiliang Software Technology Co., Ltd., Shanghai, China) to analyze each mouse's swimming patterns. Based on the recording, we measured a few key parameters: escape latency (s), number of platform crossings, time spent in the target quadrant (%), and total distance.

2.3. Network Toxicology

2.3.1. Prediction of Potential Targets of DOX and Brain Toxicology

The DOX structure and its Simplified Molecular Input Line Entry System (SMILES) number were collected from

the PubChem database (https://pubchem.ncbi.nlm.nih.gov). The potential toxic targets of DOX were predicted by the Swiss Target Prediction (http://www.swisstargetprediction.ch/), PharmMapper (https://lilab-ecust.cn/pharmmapper/index.html), and Therapeutic Target Database (https://db.idrblab.net/ttd) databases, with *Homo sapiens* selected as the species. Brain toxicology-related targets were obtained from GeneCards (https://www.genecards.org/) and OMIM (https://ngdc.cncb.ac.cn/databasecommons/database/id/109), using the keywords 'brain toxicology' and 'cognitive damage' to define the search. All predicted results were exported, and the UniProt database was used for name standardization and deduplication.

2.3.2. Construction of Protein-protein Interaction (PPI) Networks and Identification of Key Targets

The Venny website was used to identify the intersection between the targets of DOX and brain-toxicology-associated genes. The STRING database (Version 12.0) with a confidence score of 0.4 was employed to analyze the intersection genes. Subsequently, the results were visualized by using Cytoscape (version 3.9.1). The CytoHubba plug-in for Cytoscape, with Degree, Maximal Clique Centrality (MCC), and Closeness, was used to identify the top 10 key targets [19, 20].

2.3.3. Gene Ontology (GO) and Kyoto Encyclopedia of Genes and Genomes (KEGG) Analysis

The intersection genes were further analyzed using the DAVID database (https://david.ncifcrf.gov/), and *Homo sapiens* was selected. GO analysis was performed to elucidate the top 10 categories of biological processes, cellular components, and molecular functions. KEGG analysis was conducted to identify metabolic and signaling pathways, and the top 20 KEGG pathways were analyzed and presented.

2.3.4. Docking of Molecules

To analyze the binding affinities and interaction modes between DOX and the key targets, molecular docking was employed [21]. The DOX molecular structure was sourced from the PubChem database. The 3D candidate proteins of AKT1, ALB, CASP3, EGFR, ESR1, HSP90AA1, MMP9, TNF, TP53 and SRC were acquired from the PDB (https://www.rcsb.org/). After removing water molecules and replenishing polar hydrogen atoms, molecular docking based on Autodock Vina 1.2.2 (https://autodock.scripps.edu/) was performed with the grid box set as 30 Å * 30 Å * 30 Å and grid point distance set as 0.05 nm. Finally, the visualization of these results was conducted by using PyMOL.

2.4. Nissl Staining

To assess the Neuronal quantity and morphology in the cortex and hippocampus, Nissl staining was performed. Briefly, Paraffin-embedded coronal sections (5 µm thick) containing the hippocampus were dewaxed with xylene and hydrated with gradient ethanol. Then, the brain slices were stained with a cresyl violet staining solution, followed by sealing with neutral gum. Finally, the pictures were obtained under a microscope (Leica, Germany). The number of Nissl-positive cells in the cortex and the hippocampus (CA1) was counted using ImageJ (version 1.8.0).

2.5. RNA-Sequencing

For RNA sequencing analysis, the total RNA of the hippocampal tissues was collected by eliminating the genomic DNA with DNase I. The quality and concentration of the collected RNA were assessed. Subsequently, the sequencing analysis was conducted by Majorbio Bio-Pharm Biotechnology Co., Ltd (Shanghai, China) using an Illumina NovaSeq 6000. Bioinformatics analysis was performed *via* the Majorbio Cloud online platform. The differentially expressed genes (DEGs) with $|\log2\text{fold change } (\log2FC)| > 1$ and $P\text{-value} < 0.05$ between Ctrl and DOX-treated groups were obtained and subjected to GO and KEGG analysis.

2.6. Immunofluorescence Staining

Immunofluorescence staining was performed as previously described [22, 23]. Briefly, following a blocking step with normal goat serum, the slides were subsequently incubated with the primary antibodies (Table **S1**). Next, after incubation with fluorescent-labeled secondary antibody (goat anti-rabbit IgG) at 37°C for 1 h, the slices were stained with DAPI for 10 min. Finally, the images were acquired by using a fluorescence microscope (Leica, Germany).

2.7. Western Blot

The total proteins were extracted from the collected cortex and hippocampus tissues by RIPA buffer containing protease inhibitors. Protein concentrations were quantified using a BCA protein assay kit (Beyotime, China). Next, the proteins underwent electrophoresis and transmembrane electrophoresis. Afterward, blocking was performed with 5% non-fat milk for 60 min. The membranes were incubated overnight at 4°C with the primary antibodies (Table **S1**), followed by a 30-minute incubation with secondary antibodies. Finally, the bands were visualized by Tanon 5200 (Shanghai, China).

2.8. Statistical Analysis

The n values represent biological replicates and have been included in the results. All data were presented as the mean ± SEM and analyzed using Prism 8.0 software. The Shapiro-Wilk test was used to determine whether the data were normally distributed, while the Bartlett and Brown-Forsythe tests were used to test for equal variances. The nonparametric data were analyzed using the Mann-Whitney test or Kruskal-Wallis, followed by Dunn's post hoc test. Parametric data were analyzed using a t-test or a one-way ANOVA followed by Tukey's post hoc test. Data from escape latency trials in the MWM were analyzed using a two-way repeated-measures ANOVA. Based on the evaluation of assumptions (normality and sphericity) and the significance of the interaction effect, pairwise comparisons were conducted using either the t-test or Tukey's HSD test (for main effects or simple effects). *P*-value of less than 0.05 was considered statistically significant.

3. RESULTS

3.1. DOX-induced Spatial Learning and Memory Impairment in Mice

To clarify the detrimental effects of DOX on spatial learning and memory, mice were treated with a single intraperitoneal injection of 25 mg/kg DOX, which is equivalent to the maximum single therapeutic dose used in clinical settings. Then, the MWM test was conducted (Figs. **1A**, **B**). As shown in Figs. (**1C-E**), the escape latency of the mice in each group decreased over successive days, and the DOX-treated mice took longer to escape and find the hidden platform compared to the Ctrl group on days 3 (n = 8 mice per group, P = 0.016), 4 (n = 8 mice per group, P = 0.028), and 5 (n = 8 mice per group, P = 0.0007). Furthermore, the results of the spatial probe test revealed that the Ctrl group, but not the DOX-treated group, showed a clear bias toward the target quadrant (Fig. **1D**). The mice in the DOX-treated group exhibited less residence time in the target quadrant (n = 8 mice per group, P < 0.0001) and fewer platform crossings (n = 8 mice per group, P < 0.0001) (Figs. **1F**, **G**). However, the two groups showed comparable swimming speed (n = 8 mice per group, P = 0.973) and total distance (n = 8 mice per group, P = 0.653), with no significant differences (Figs. **1H**, **I**).

3.2. DOX Treatment Resulted in Neuronal Loss and Synapse-related Protein Decline in the Brain

Given that DOX treatment caused cognitive impairment in mice, we further investigated its effects on neuronal and synaptic loss in brain tissues. Firstly, the results of Nissl staining showed that DOX treatment markedly reduced the number of positive Nissl bodies in both the cortex (n = 5 mice per group, P = 0.001) and hippocampus (n = 5 mice per group, P = 0.002) (Figs. **2A**, **B**). Meanwhile, the mice in the DOX group exhibited fewer NeuN-positive neurons than those in the Ctrl group in the cortex (n = 5 mice per group, P = 0.0001) and hippocampus (n = 5 mice per group, P < 0.0001) (Figs. **2C**, **D**). These data indicated that DOX treatment resulted in neuronal loss in the cortex and hippocampus. As we know, Microtubule-associated protein 2 (MAP2)

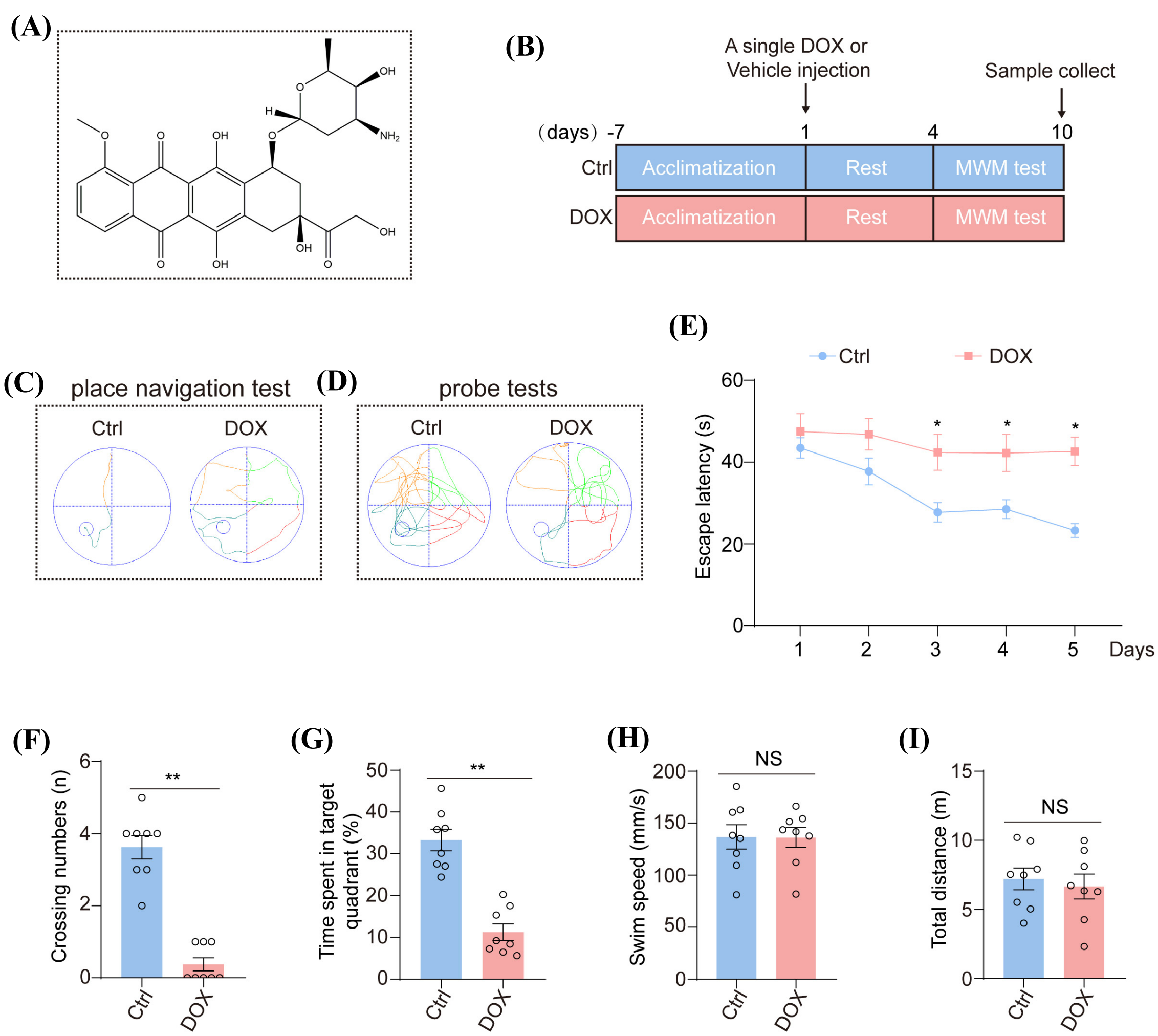

Fig. (1). DOX treatment induced cognitive damage in mice. (**A**) The chemical structure of DOX. (**B**) The flowchart of the DOX treatment protocol. (**C**) Representative swim paths during the place navigation test. (**D**) Representative swim trajectories during the probe trial. (**E**) Effect of DOX on latency during the training phase of the MWM in mice. (**F**) Effect of DOX on crossing numbers of the target area. (**G**) Effect of DOX on time spent in the target quadrant. (**H**) Effect of DOX on the swim speed of mice. (**I**) Effect of DOX on the total distance of mice. $^{*}P$ < 0.05, $^{**}P$ < 0.01. NS, no difference. (*A higher resolution/colour version of this figure is available in the electronic copy of the article*).

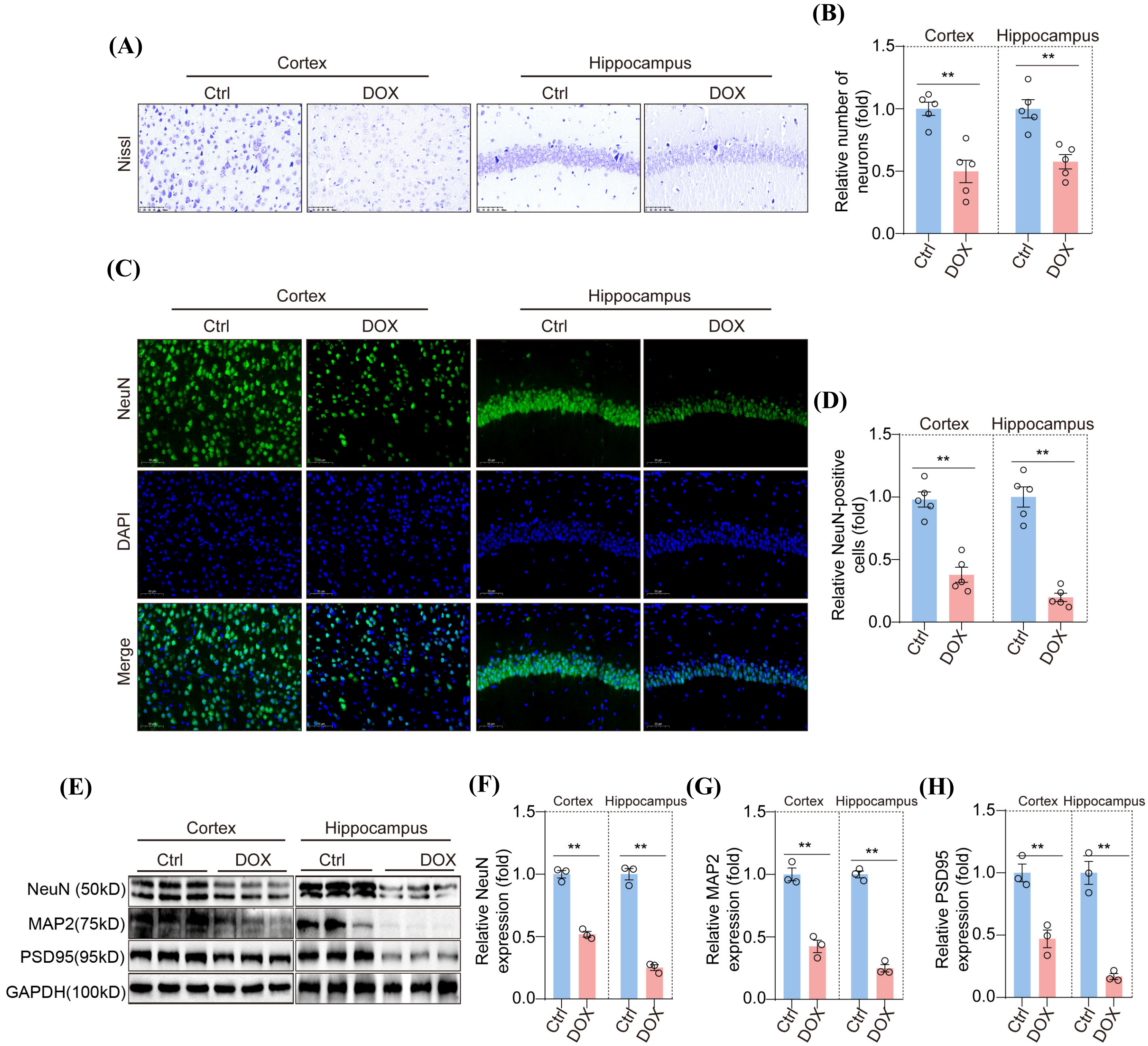

Fig. (2). DOX treatment induced neuronal and synaptic loss in mice. (**A**, **B**) Nissl staining of the cortex and hippocampus tissues. (**C**, **D**) Representative images and quantitative analysis of NeuN-positive cells in the cortex and hippocampus of mice. (**E-H**) Western blotting and densitometry analysis of NeuN, MAP2, and PSD95 in the cortex and hippocampus. $^{*}P < 0.05$, $^{**}P < 0.01$ *vs*. indicated group. (*A higher resolution/colour version of this figure is available in the electronic copy of the article*).

is primarily localized in neuronal dendrites, and its deficiency can be considered an indicator of imminent neuronal death [24]. Postsynaptic density protein 95 (PSD95) is essential for the appropriate formation and maintenance of excitatory synapses. A low PSD95 level has been reported to be associated with cognitive impairment [25]. Therefore, the levels of these two synapse-related proteins in the cortex and hippocampus of mice were examined. Consistently, compared with the Ctrl group, the DOX group possessed significantly lower levels of NeuN (cortex: $n = 3$ mice per group, $P = 0.0002$; hippocampus: $n = 3$ mice per group, $P = 0.0001$), MAP2 (cortex: $n = 3$ mice per group, $P = 0.001$; hippocampus: $n = 3$ mice per group, $P < 0.0001$), and PSD95 (cortex: $n = 3$ mice per group, $P = 0.006$; hippocampus: $n = 3$ mice per group, $P = 0.001$) (Figs. **2E-H**). In addition, the immunoblotting results showed that DOX treatment markedly decreased BDNF expression in the cortex ($n = 3$ mice per group, $P = 0.0002$) and hippocampus ($n = 3$ mice per group, $P = 0.0016$) (Fig. **S1A-B**). All these data suggested that DOX treatment induced neuronal loss and a decline in synapse-related proteins in the brains of mice.

3.3. Characterization of DOX-induced Cognitive Damage *via* Network Toxicology and RNA-Seq Analysis

To gain deeper mechanistic insights into DOX-induced neurotoxicity, we integrated network toxicology and the transcriptomes of hippocampus tissues from the Ctrl and

DOX groups. As shown in Table **S2**, the structure and properties of DOX were retrieved from PubChem. Then, SMILES numbers for DOX were submitted to Swiss Target Prediction, PharmMapper, and the Therapeutic Target Database, yielding 439 potential DOX targets. In addition, 3583 genes associated with brain toxicity and cognitive damage were collected from the GeneCards and OMIM databases. After deduplication and integration of these genes, a total of 241 intersection genes were identified as potential targets of DOX-induced cognitive damage (Fig. **3A**). Next, we imported 241 potential genes into the STRING database and constructed a PPI network using Cytoscape. As illustrated in Fig. (**S2**), the network contained 240 nodes and 3765 edges, with an average node degree of 13.6. The 241 collective targets were subsequently used to construct GO and KEGG analyses using the DAVID database.

The top 20 GO enrichment terms were selected and presented in Fig. (**S3**). The results indicated that the targets of DOX-induced cognitive damage were involved in regulating protein phosphorylation, phosphatidylinositol 3-kinase/ protein kinase B signaling, enzyme binding, protein kinase activity, and protein phosphatase binding. The top 20 terms of KEGG pathway enrichment were selected and displayed in Fig. (**3B**). The targets of DOX-induced brain damage were primarily enriched in pathways related to cancer, lipids and atherosclerosis, proteoglycans in cancer, hepatitis B, and fluid shear stress and atherosclerosis. Mechanistically, pathway analysis indicated that PI3K/AKT1, MAPK, FOXO, AGE-RAGE, and vascular endothelial growth factor (VEGF) signaling pathways may play crucial roles in DOX-mediated neurotoxicity. Subsequently, the PPI network of key targets was constructed and revealed that AKT serine/threonine kinase 1 (AKT1), Albumin (ALB), Caspase 3 (CASP3), Epidermal growth factor receptor (EGFR), Estrogen receptor 1 (ESR1), Heat shock protein 90 alpha family class a member 1 (HSP90AA1), Matrix metalloproteinase 9 (MMP9), Tumor necrosis factor (TNF), Tumor protein P53 (TP53), and SRC proto-oncogene (SRC) are the top 10 key targets (Fig. **3C**). Further, we explored whether there were interactions between DOX and key proteins (AKT1, ALB, CASP3, EGFR, ESR1, HSP90AA1, MMP9, TNF, TP53, and SRC). The results of molecular docking uncovered that nine key proteins (AKT1, ALB, CASP3, EGFR, ESR1, HSP90AA1, MMP9, TNF, and TP53) exhibited strong binding affinities with DOX (Fig. **3D** and Table **S3**), while no interaction between DOX and SRC was observed. The visualizations of the lowest binding energies between DOX and key targets were generated by using PyMOL (Fig. **3D**). The PCA results showed that the DOX group was separated from the Ctrl group, suggesting that DOX intervention markedly altered the transcriptional profiles (Fig. **3E**). Further analysis found that 644 genes were significantly and differentially expressed in the two groups ($P < 0.05$ and $|\log_2$fold change $(\log_2FC)| > 1$), while DOX treatment markedly upregulated 379 gene levels and downregulated 265 gene expression (Figs. **3F**, **G**). Additionally, the GO enrichment results showed that the 644 differentially expressed genes were substantially related to developmental process, extracellular space and region, ion binding, and signaling receptor binding (Fig. **3H**). The KEGG analysis revealed that these differential genes were enriched in the Neuroactive ligand-receptor interaction, PPAR signaling pathway, VEGF signaling pathway, PI3K-Akt signaling pathway, and MAPK signaling pathway (Fig. **3I**).

3.4. Effects of DOX on the PI3K-AKT-VEGF Signaling Pathway in Mice

To comprehensively clarify the underlying mechanism of DOX-caused cognitive impairment, a joint analysis of network toxicology and transcriptome was performed. Ultimately, four essential pathways, including the PI3K-AKT signaling pathway, the VEGF signaling pathway, the MAPK signaling pathway, and Proteoglycans in cancer, were common to both the network toxicology and transcriptome and then identified as the potential pathways involved in DOX-caused cognitive impairment (Fig. **4A**). Considering the good affinity between DOX and AKT1, TP53, EGFR, MMP9, as well as HSP90AA1, we speculated that the PI3K-AKT-VEGF signaling pathway might be a critical signaling pathway involved in DOX-induced cognitive damage. Further immunoblotting showed that DOX treatment markedly increased the p-PI3K (cortex: $n = 3$ mice per group, $P < 0.0001$; hippocampus: $n = 3$ mice per group, $P = 0.002$), p-AKT (cortex: $n = 3$ mice per group, $P = 0.0007$; hippocampus: $n = 3$ mice per group, $P = 0.0002$), and VEGFA (cortex: $n = 3$ mice per group, $P = 0.0002$; hippocampus: $n = 3$ mice per group, $P < 0.0001$) levels in the cortex and hippocampus tissues, indicating that DOX treatment activated the PI3K-AKT-VEGF pathway (Figs. **4B-E**). As we know, VEGF mainly originates from astrocytes in the brain. Therefore, we detected the expression of p-AKT$^+$/GFAP$^+$ and VEGFA$^+$/GFAP$^+$ double-positive cells by immunofluorescence. Consistently, immunofluorescence staining showed that the number of p-AKT$^+$/GFAP$^+$ ($n = 5$ mice per group, $P < 0.0001$) and VEGFA$^+$/GFAP$^+$ ($n = 5$ mice per group, $P < 0.0001$) double-positive cells was increased after DOX treatment (Figs. **4F-I**). Taken together, these data indicate that activation of the PI3K-AKT-VEGF pathway in astrocytes may be involved in DOX-induced cognitive impairment.

3.5. Effect of DOX on BBB Permeability in the Brain of Mice

The VEGF signaling pathway is crucial for regulating angiogenesis and vascular permeability, and excessive VEGF expression in pathological states can lead to BBB damage [26]. Therefore, we speculated that DOX may impair the permeability of the tBBB. As shown in Fig. (**S4**), EB extravasation was absent in the brains of Ctrl mice but was markedly increased following DOX treatment, indicating BBB impairment. ZO-1 and Occludin, two BBB tight junction proteins, are crucial for BBB permeability. As shown in Figs. (**5A-C**), our findings suggested that the expression of ZO-1 (cortex: $n = 3$ mice per group, $P = 0.0005$; hippocampus: $n = 3$ mice per group, $P = 0.0001$) and Occludin (cortex: $n = 3$ mice per group, $P = 0.0009$; hippocampus: $n = 3$ mice per group, $P = 0.0006$) was remarkably downregulated in the cortex and hippocampus of DOX-treated mice in comparison to the Ctrl group. Consistently, immunofluorescence

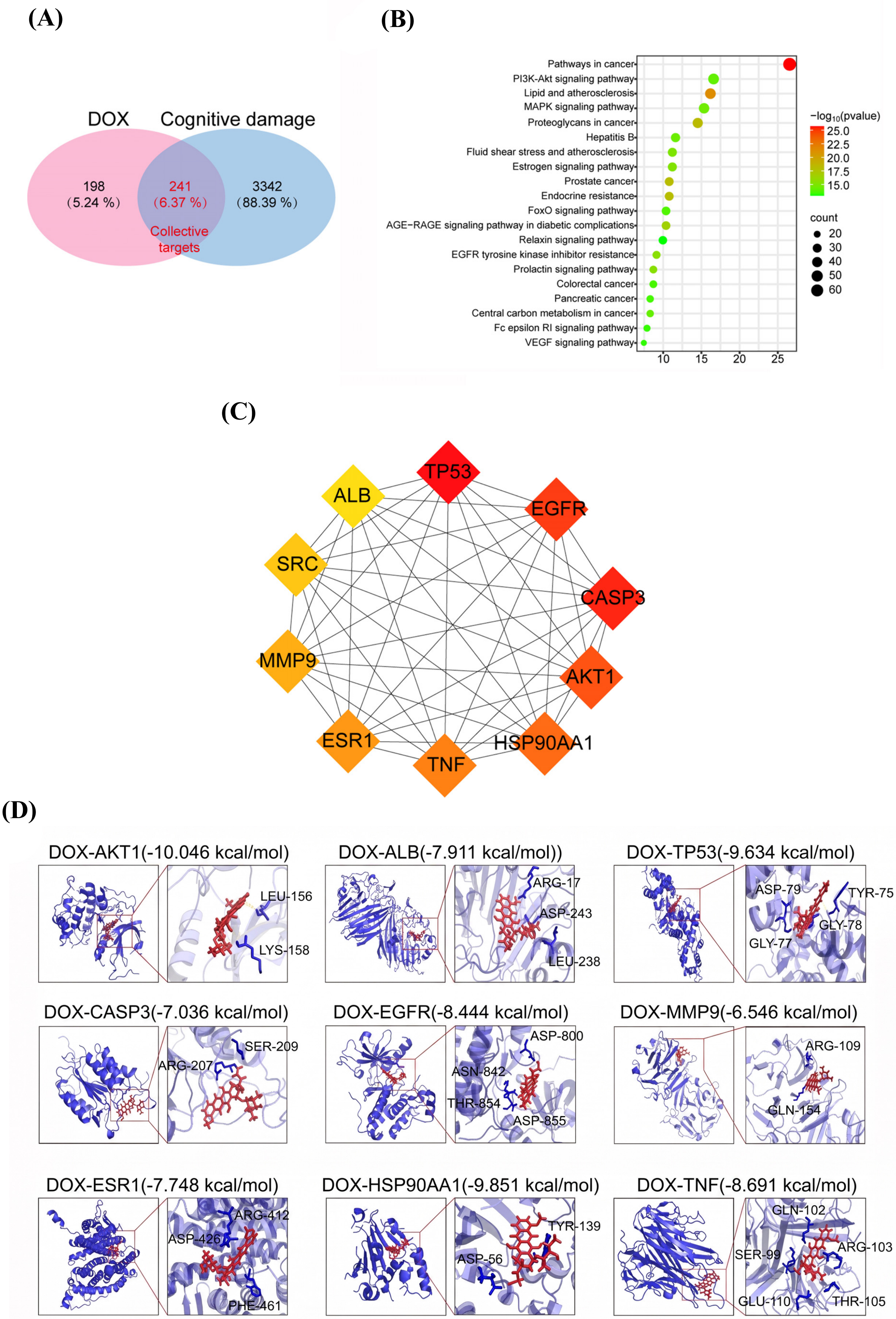
(A)
DOX
Cognitive damage
198
(5.24 %)
241
(6.37 %)
3342
(88.39 %)
Collective targets
(B)
Pathways in cancer
PI3K-Akt signaling pathway
Lipid and atherosclerosis
MAPK signaling pathway
Proteoglycans in cancer
Hepatitis B
Fluid shear stress and atherosclerosis
Estrogen signaling pathway
Prostate cancer
Endocrine resistance
FoxO signaling pathway
AGE−RAGE signaling pathway in diabetic complications
Relaxin signaling pathway
EGFR tyrosine kinase inhibitor resistance
Prolactin signaling pathway
Colorectal cancer
Pancreatic cancer
Central carbon metabolism in cancer
Fc epsilon RI signaling pathway
VEGF signaling pathway
10 15 20 25
−log10(pvalue)
25.0
22.5
20.0
17.5
15.0
count
20
30
40
50
60
(C)
TP53
ALB
EGFR
SRC
CASP3
MMP9
AKT1
ESR1
TNF
HSP90AA1
(D)
DOX-AKT1(-10.046 kcal/mol)
LEU-156
LYS-158
DOX-ALB(-7.911 kcal/mol))
ARG-17
ASP-243
LEU-238
DOX-TP53(-9.634 kcal/mol)
ASP-79
TYR-75
GLY-78
GLY-77
DOX-CASP3(-7.036 kcal/mol)
SER-209
ARG-207
DOX-EGFR(-8.444 kcal/mol)
ASP-800
ASN-842
THR-854
ASP-855
DOX-MMP9(-6.546 kcal/mol)
ARG-109
GLN-154
DOX-ESR1(-7.748 kcal/mol)
ARG-412
ASP-426
PHE-461
DOX-HSP90AA1(-9.851 kcal/mol)
TYR-139
ASP-56
DOX-TNF(-8.691 kcal/mol)
GLN-102
ARG-103
SER-99
GLU-110
THR-105

(Fig. 3) Contd….

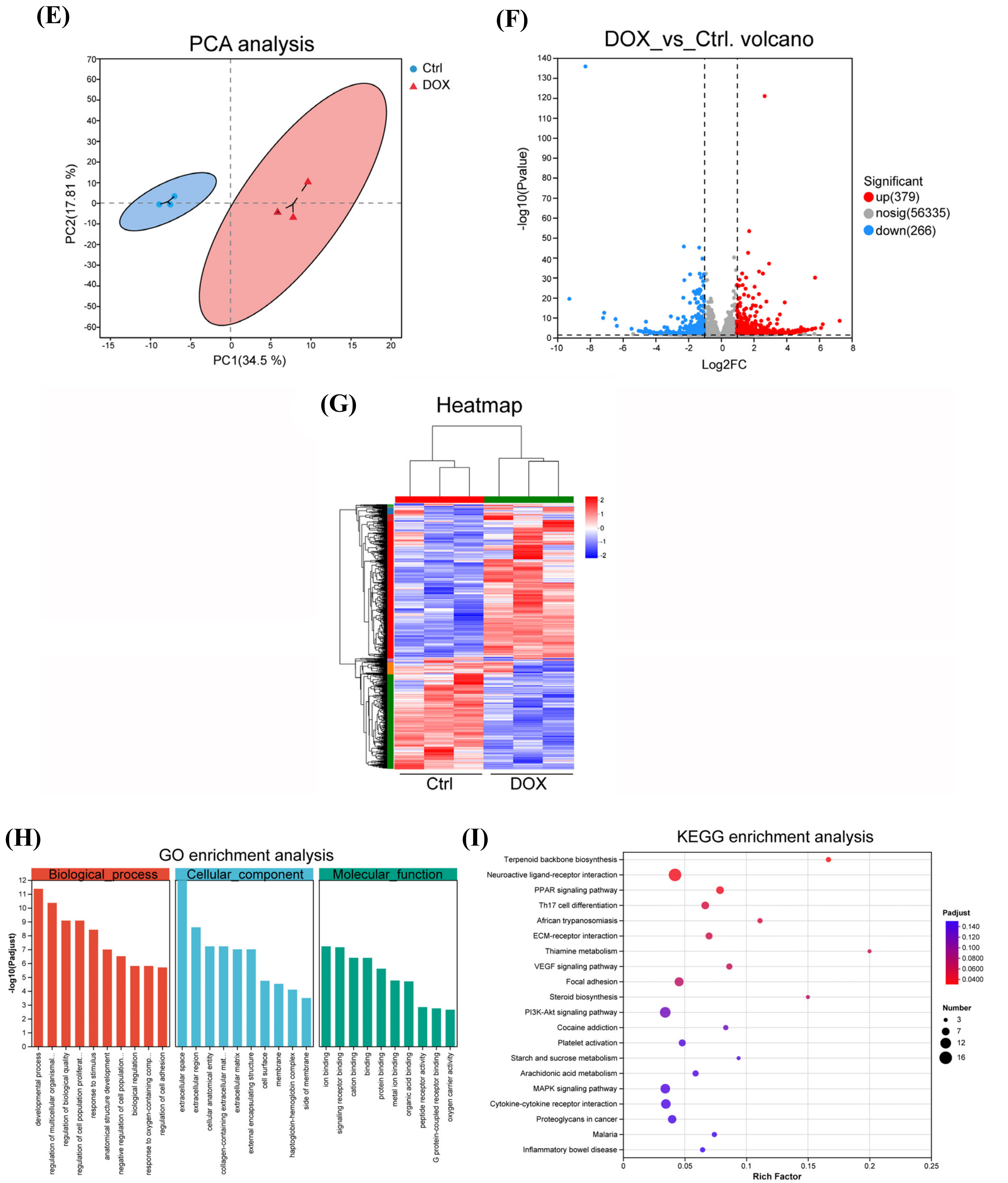

Fig. (3). Identification of the key molecular targets and pathogenic mechanisms underlying DOX-induced cognitive impairment. (**A**) A Venn diagram showing the intersection of genes of DOX and cognitive damage. (**B**) The KEGG pathway analysis bubble diagram. (**C**) The top 10 key targets were screened by using CytoHubba. (**D**) Binding models between key targets and DOX. (**E**) PCA analysis of the transcriptomic data from RNA-seq. (**F**) Volcano plot of differentially expressed genes. The blue dots represent the genes decreased by DOX, while the red dots represent the genes upregulated by DOX. (**G**) Heat maps were produced for DEGs between the Ctrl and DOX groups. (**H**) GO enrichment analysis of the regulated DEGs between the Ctrl and DOX groups. (**I**) Bubble Diagram of the 20 significantly enriched KEGG pathways between the Ctrl and DOX groups. (*A higher resolution/colour version of this figure is available in the electronic copy of the article*).

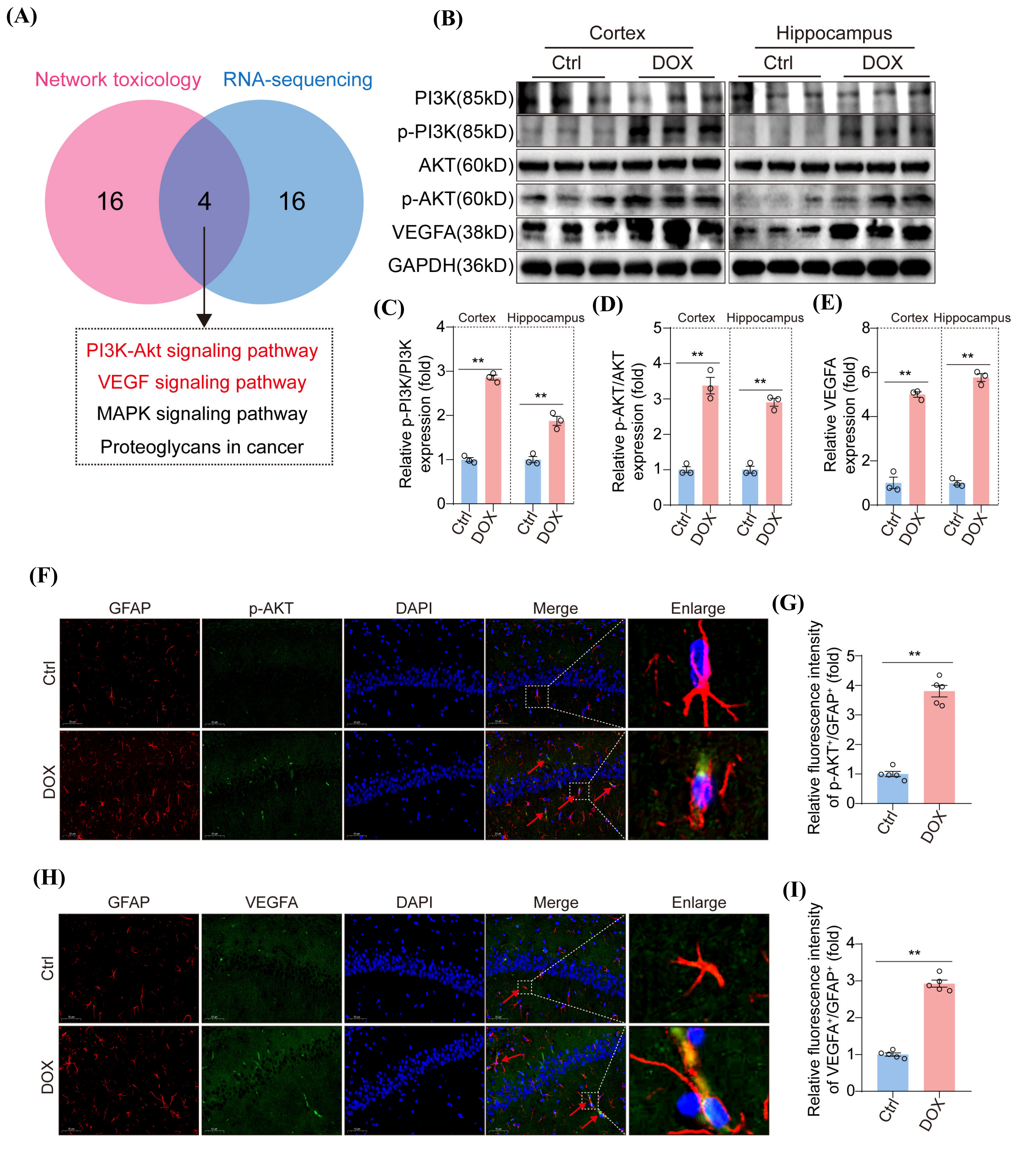

Fig. (4). (**A**) Venn plot showing the strategy for screening the essential pathways both enriched by network toxicology and transcriptome. (**B-E**) Representative Western blotting images of p-PI3K, PI3K, p-AKT, AKT, and VEGFA in the cortex and hippocampus of mice. (**F-G**) Immunofluorescence co-localization staining and quantitation of p-AKT and GFAP in the cortex and hippocampus of mice. (**H-I**) Immunofluorescence co-localization staining and quantitation of VEGFA and GFAP in the cortex and hippocampus of mice. *$P < 0.05$, **$P < 0.01$ *vs*. indicated group. (*A higher resolution/colour version of this figure is available in the electronic copy of the article*).

staining also revealed that the fluorescent density of ZO-1$^+$/CD31$^+$ positive cells (cortex: $n = 5$ mice per group, $P < 0.0001$; hippocampus: $n = 5$ mice per group, $P < 0.0001$) and OCC$^+$/CD31$^+$ (cortex: $n = 5$ mice per group, $P < 0.0001$; hippocampus: $n = 5$ mice per group, $P < 0.0001$) positive cells in the cerebral cortex and hippocampus of the DOX-treated group was significantly lower than that of the Ctrl group (Figs. **5D-G**). These data suggested the possible disruption of the BBB in the brain following DOX exposure.

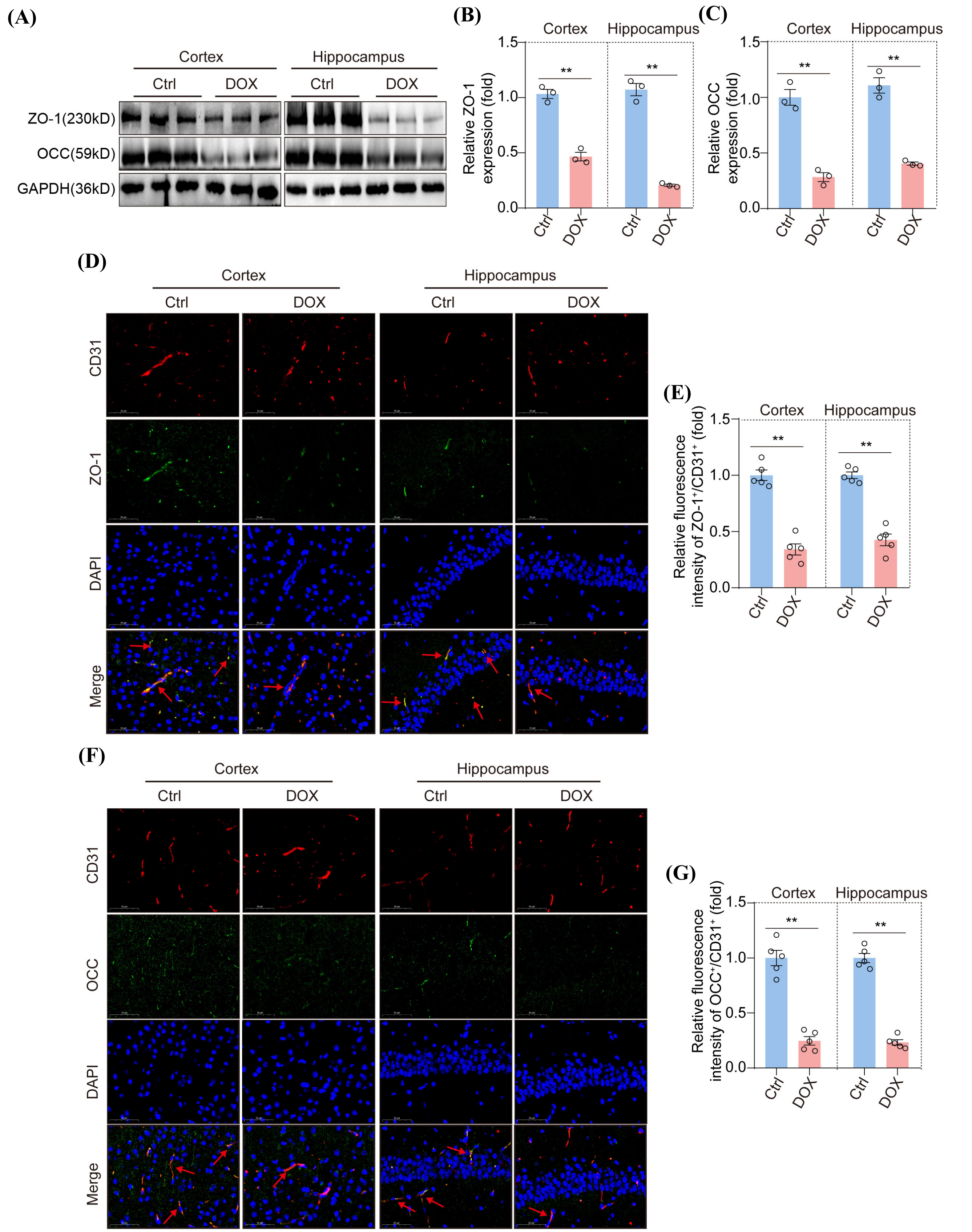

Fig. (5). Effect of DOX on the barrier function in mice. (**A-C**) Western blotting and densitometry analysis of ZO-1 and OCC in the cortex and hippocampus of mice. (**D**, **E**) Immunofluorescence co-localization staining and quantitation of ZO-1 and CD31 in the cortex and hippocampus of mice. (**F**, **G**) Immunofluorescence co-localization staining and quantitation of OCC and CD31 in the cortex and hippocampus of mice. *P < 0.05, **P < 0.01 *vs*. indicated group. (*A higher resolution/colour version of this figure is available in the electronic copy of the article*).

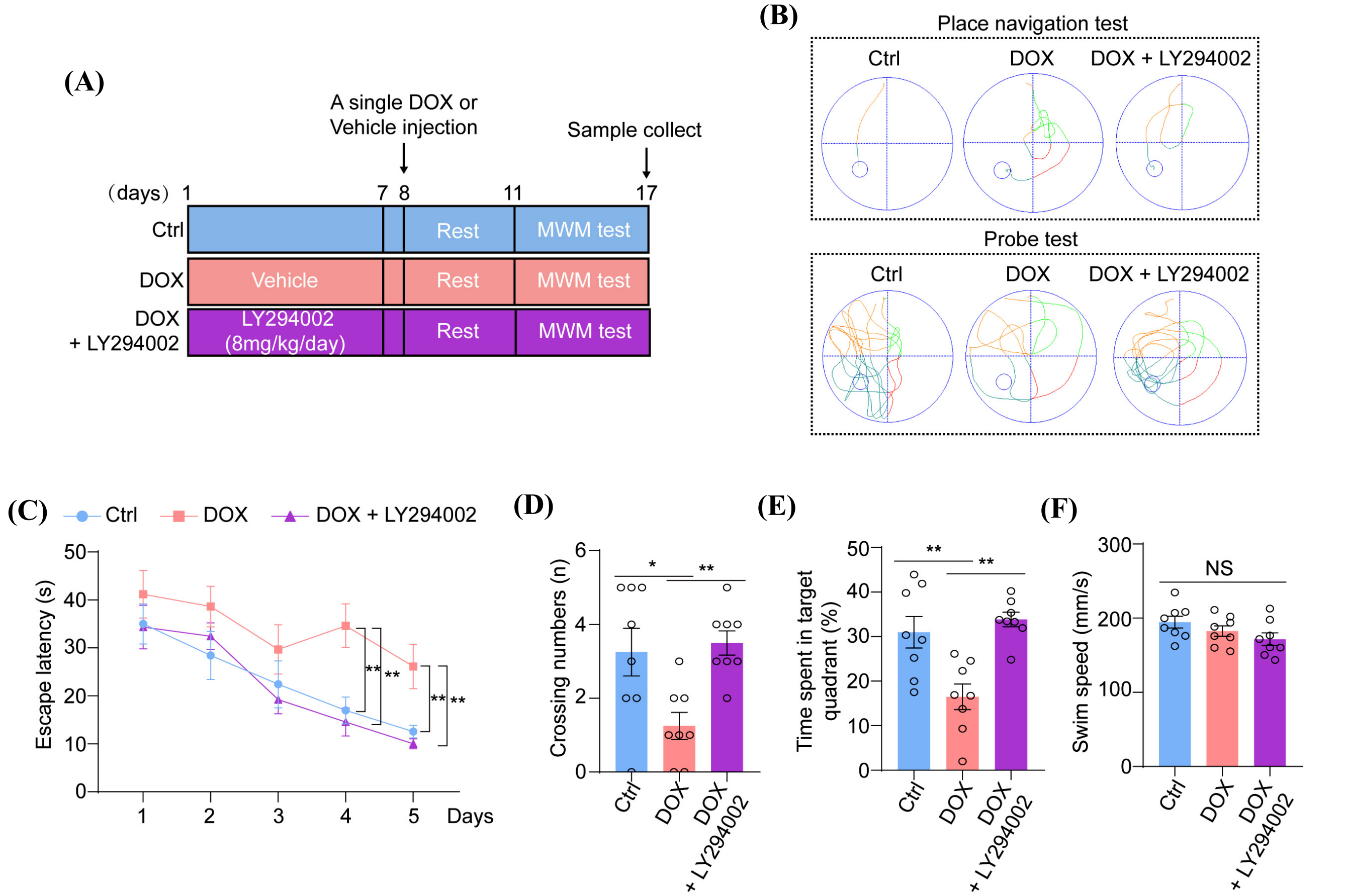

Fig. (6). LY294002 alleviated DOX-induced cognitive damage. (**A**) The intervention flowchart of LY294002 and DOX. (**B**) Representative swim paths during the place navigation test and the probe test. (**C**) Effect of DOX on latency during the training phase of the MWM in mice. (**D**) Effect of DOX on crossing numbers of the target area. (**E**) Effect of DOX on time spent in the target quadrant. (**F**) Effect of DOX on the swim speed of mice. $^{*}P < 0.05$, $^{**}P < 0.01$ *vs*. indicated group. (*A higher resolution/colour version of this figure is available in the electronic copy of the article*).

3.6. PI3K Inhibitor LY294002 Treatment Alleviated DOX-induced Cognitive Damage

The above results have suggested that DOX treatment induced cognitive deficits, disrupted BBB permeability, and activated the PI3K-AKT-VEGF pathway. Previous studies have verified the neuroprotective effects of LY294002 in animal models [18]. Therefore, we asked whether inhibition of the PI3K-AKT pathway by LY294002 could alleviate DOX-induced cognitive damage (Fig. **6A**). As shown in Figs. (**6B**, **C**), the escape latency of mice treated with DOX was significantly increased on days 4 (n = 8 mice per group, P = 0.0058) and 5 (n = 8 mice per group, P = 0.043). However, administration of LY294002 markedly reversed the DOX-induced increase in escape latency(days 4: n = 8 mice per group, P = 0.0014; days 5: n = 8 mice per group, P = 0.0131). In addition, although no statistically significant differences in swimming speed were found among the three groups, mice in the DOX + LY294002 group exhibited higher platform crossing numbers (n = 8 mice per group, P = 0.0075) and longer durations in the target quadrant (n = 8 mice per group, P = 0.0007) than mice in the DOX-treated group (Figs. **6D-F**). These data suggested that LY294002 treatment markedly ameliorated learning and memory dysfunctions caused by DOX in mice.

3.7. LY294002 Attenuated BBB Disruption and Prevented Neuronal Loss in DOX-treated Mice

Finally, LY294002 was identified as improving barrier function and preventing neuronal loss in DOX-triggered CRCI models. As shown in Fig. **S5**, LY294002 treatment significantly reduced the DOX-induced accumulation of EB in the brain. As presented in Figs. (**7A-D**), DOX treatment significantly upregulated the number of p-AKT^{+}/GFAP^{+} and VEGFA^{+}/GFAP^{+} positive cells in the hippocampus, which were markedly attenuated by treatment with LY294002 (p-AKT^{+}/GFAP^{+}: n = 5 mice per group, P < 0.0001; VEGFA^{+}/GFAP^{+}: n = 5 mice per group, P = 0.0003). Furthermore, immunofluorescence staining showed that there was a significant decrease in ZO-1^{+}/CD31^{+}-positive cells in the brain in the DOX mice, and LY294002 significantly increased the ZO-1^{+}/CD31^{+}-positive cells in the brain (n = 5 mice per group, P = 0.0335) (Figs. **7E** and **F**). Additionally, we showed that the LY294002 intervention significantly reversed DOX-triggered downregulation of NeuN-positive cells in the hippocampus of mice (n = 5 mice per group, P = 0.0059) (Figs. **7G** and **H**). Consistently, as shown in Fig. (**7I**), immunoblotting results demonstrated that DOX treatment upregulated the protein levels of p-PI3K, p-AKT, and VEGFA, and these effects were attenuated by LY294002

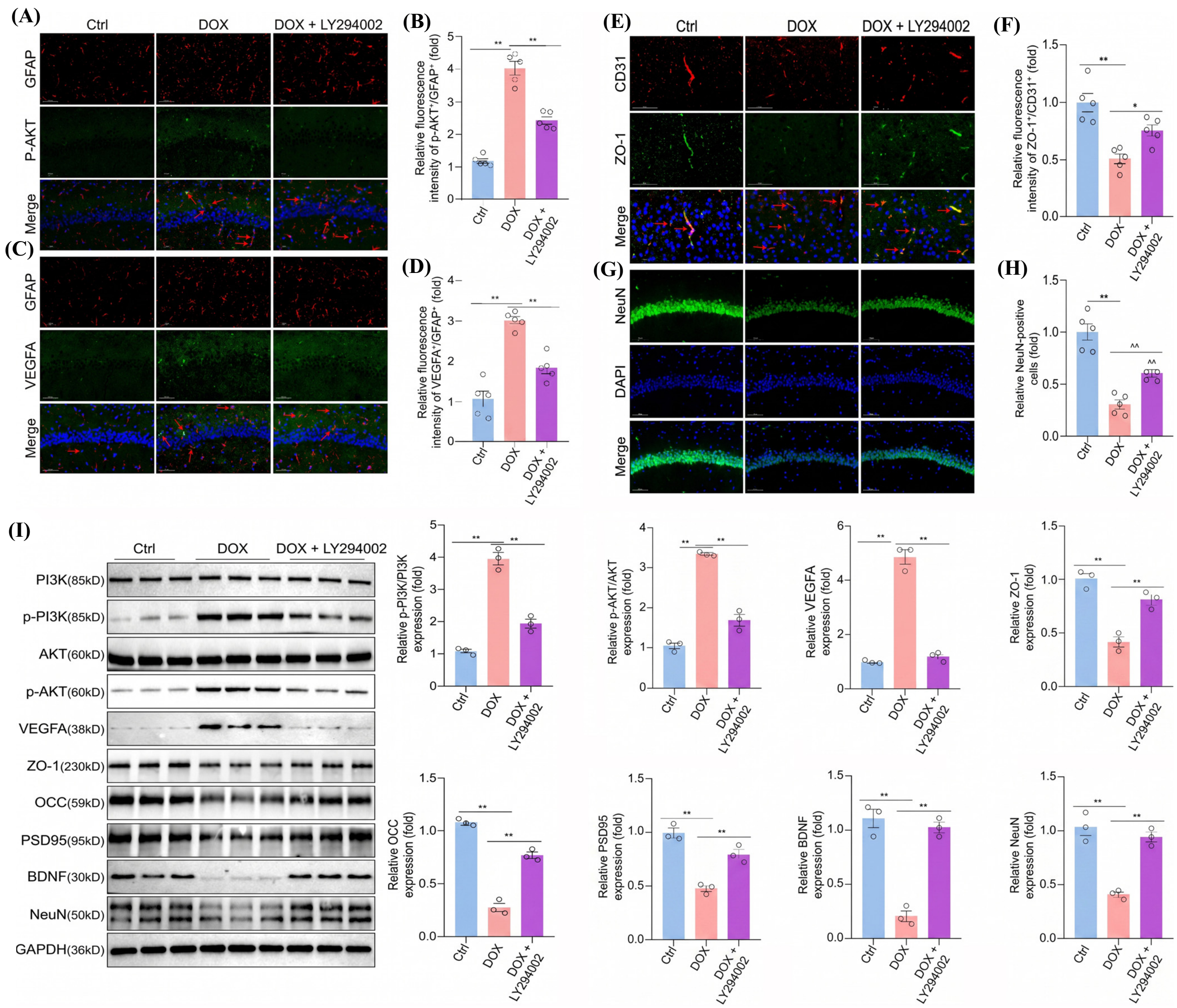

Fig. (7). The PI3K-AKT-VEGF pathway was downregulated, and LY294002 improved barrier function *in vivo*. (**A**, **B**) Immunofluorescence co-localization staining of p-AKT and GFAP. (**C**, **D**) Immunofluorescence co-localization staining of VEGFA and GFAP. (**E**, **F**) Immunofluorescence co-localization staining of ZO-1 and CD31. (**G**, **H**) The number of NeuN-positive cells in hippocampal tissue. (**I**) Western blotting and densitometry analysis of p-PI3K, p-AKT, VEGFA, ZO-1, Occludin, PSD95, BDNF, and NeuN in the hippocampus of mice. $^*P < 0.05$, $^{**}P < 0.01$ *vs*. indicated group. (*A higher resolution/colour version of this figure is available in the electronic copy of the article*).

(p-PI3K: $n = 3$ mice per group, $P = 0.0001$; p-AKT: $n = 3$ mice per group, $P < 0.0001$; VEGFA: $n = 3$ mice per group, $P < 0.0001$). Conversely, the levels of ZO-1, Occludin, PSD95, BDNF, and NeuN were significantly decreased in the DOX group compared to the Ctrl group, and treatment with the PI3K inhibitor LY294002 reversed these changes (ZO-1: $n = 3$ mice per group, $P = 0.003$; Occludin: $n = 3$ mice per group, $P < 0.0001$; PSD95: $n = 3$ mice per group, $P = 0.0032$; BDNF: $n = 3$ mice per group, $P = 0.0002$; NeuN: $n = 3$ mice per group, $P = 0.0011$). Collectively, these results indicated that LY294002 improved barrier function and prevented neuronal loss in DOX-treated mice.

4. DISCUSSION

Doxorubicin (DOX), an anthracycline antibiotic derived from the genus Streptomyces, is widely used as a chemotherapeutic agent in the treatment of many types of cancer, including lung, leukemia, and breast cancer [27]. Despite its potent therapeutic actions, DOX also induces cytotoxicity in various organs, including the heart, brain, liver, and kidneys [28, 29]. Previous studies have reported that patients receiving DOX treatment across all age groups exhibited declines in cognitive performance on cognitive assessments [9]. However, the potential mechanisms underlying DOX-mediated neurotoxicity have not been thoroughly investigated. In the current study, an integrated strategy combining network toxicology, transcriptomic analysis, and *in vivo* experiments was used to elucidate the direct neurotoxic molecular mechanism underlying doxorubicin (DOX)-induced cognitive impairment.

Network toxicology is integral to predicting toxicological outcomes and elucidating the underlying mechanisms of

toxicity. For example, a previous study utilizing network toxicology demonstrated that prostate injury induced by 4,4-sulfonyl diphenol might be associated with apoptosis and inflammatory response [30]. Ge *et al*. employed network toxicology to elucidate the hepatotoxicity of aflatoxin B1, uncovering the involvement of apoptosis, the PI3K-AKT1 signaling pathway, and the EGFR-ERK signaling pathway [31]. In the current study, we employed network toxicology to identify potential targets associated with DOX-triggered cognitive damage, and identified 241 targets. Identifying key targets is essential for elucidating network regulation, and the MCC algorithm has been considered a reliable method for predicting them. In the current study, TP53, AKT1, ESR1, ALB, TNF, CASP3, HSP90AA1, EGFR, SRC, and MMP9, which exhibited the highest MCC values, were identified as the central targets associated with DOX-induced cognitive damage. Among these key targets, TP53, a vital tumor suppressor, is generally recognized to regulate cell cycle control and DNA repair [32]. ALB is critical for the AKT1 and EGFR signaling cascades in maintaining intracellular homeostasis. ESR1 is associated with synaptogenesis, neurogenesis, and neurotransmitter function. In addition, TNF contributes to neuroinflammatory responses, CASP3 functions as the executor of apoptosis, and HSP90AA1 is regarded as a pivotal immune key gene in synapse function and mild cognitive impairment [33]. MMP9 serves as a critical mediator in immune dysregulation and neuroinflammatory processes, concurrently exacerbating BBB breakdown. Therefore, these findings suggest that DOX-triggered cognitive damage might involve various pathways, including neuroinflammatory responses, apoptosis, synapse dysfunction, and blood-brain barrier disruption.

In network toxicological analysis, enrichment analysis is considered a key strategy for dissecting the potential molecular mechanism. In this study, GO enrichment analysis revealed that regulation of protein phosphorylation, phosphatidylinositol 3-kinase/protein kinase B signal transduction, protein kinase activity, and protein phosphatase binding were the primary biological processes involved in DOX-induced brain damage. The KEGG analysis indicated that the neurotoxic effects of DOX were associated with various human diseases, including cancers, lipid metabolism, proteoglycans in cancer, hepatitis B, and atherosclerosis. In addition, pathway analysis revealed that the PI3K-AKT1 signaling pathway is the primary pathway for DOX-induced cognitive damage, while the MAPK, AGE-RAGE, FOXO, and VEGF signaling pathways may also play crucial roles in DOX-mediated cognitive damage. Molecular docking is widely used to explore the potential binding of toxic molecules to protein targets. As expected, DOX binds to potential neurotoxic targets (including TP53, AKT1, ESR1, ALB, TNF, CASP3, HSP90AA1, EGFR, and MMP9), thereby supporting our network toxicology predictions.

To further investigate the mechanism of DOX-induced cognitive damage, a single DOX dose was administered to mice to establish the model, and spatial learning and memory were assessed. Our study demonstrated that DOX significantly impaired spatial learning and memory in the MWM test. Furthermore, DOX treatment resulted in neuronal loss and a decline in synapse-related proteins in both the cortex and the hippocampus of mice. In addition, given that the network toxicological analysis relies on existing literature, therefore, in our study, we also conducted transcriptome analysis to predict potential mechanisms underlying DOX-caused cognitive impairment. KEGG analysis suggested that DOX mainly affected the metabolic pathways, PPAR signaling pathway, VEGF signaling pathway, PI3K-AKT signaling pathway, and MAPK signaling pathway. By integrating network toxicology, transcriptomic analysis, and molecular docking, the PI3K-AKT-VEGF pathway was speculated to exert an essential role in DOX-induced cognitive damage.

As we know, VEGF serves as a critical mediator of angiogenesis and vascular permeability and is closely associated with blood-brain barrier dysfunction. Previously, the overproduction of VEGF was found to facilitate angiogenesis and induce BBB disruption [34]. Matsuno *et al*. also reported that the upregulation of VEGF was positively associated with BBB dysfunction and stress-induced depression [35]. Blocking the VEGF-A signaling pathway could reduce BBB breakdown and alleviate neurologic deficits in multiple sclerosis [36]. The PI3K-AKT axis functions as an intracellular heterodimer to regulate cellular biological function through a series of cascading reactions. The PI3K-AKT pathway could stabilize BBB integrity by regulating tight junction protein expression in stroke and neurodegenerative diseases [18, 37, 38]. As we know, increased BBB permeability is closely associated with cognitive deficits and neuronal loss. For instance, barrier deficiencies exacerbated neuronal loss and aggravated sepsis-mediated encephalopathy [39]. Hu and colleagues demonstrated that melatonin can alleviate anesthesia- or surgery-triggered loss of synapse-associated proteins in mice by improving BBB permeability [40]. Improving BBB permeability could help mitigate MCAO/R-induced neuronal loss and cerebral injury [41]. Interestingly, our study showed that DOX increased PI3K phosphorylation, promoted AKT1 phosphorylation, and increased VEGF levels in the cortex and hippocampus.

Furthermore, our results also showed that DOX downregulated the levels of tight junction proteins (ZO-1 and Occludin). Previously, it has been reported that peripheral administration of a specific PI3K pathway inhibitor, LY294002, can effectively inhibit PI3K-AKT pathway activation in brain tissues *in vivo* animal models [18, 42]. Notably, we also verified that treatment with the PI3K inhibitor LY294002 could improve DOX-induced cognitive damage. Additionally, LY294002 can inhibit PI3K-AKT-VEGF pathway activation in astrocytes, thereby attenuating DOX-induced disruption of the BBB and neuronal cell loss. Collectively, our study confirmed that DOX exerts neurotoxic effects by increasing BBB permeability via the activation of the PI3K-AKT-VEGF pathway.

5. LIMITATIONS

Despite providing a comprehensive analysis of DOX-induced neurotototoxicity, this study has several limitations. First, the toxicity of DOX can be categorized as acute, subacute, or chronic, with acute toxic effects typically arising 2 to 3 days after DOX administration [43]. In the current study, a single dose of DOX was administered to investigate the neurotoxic effects of acute doxorubicin treatment. Nevertheless, although the dosage used in our study is equivalent

to the maximum single therapeutic dose used clinically, patients with tumors generally receive multiple low-dose DOX treatments in clinical practice. Thus, the mechanisms of chronic DOX neurotoxicity warrant further investigation. Second, although male mice are the most commonly used mouse models in animal experiments, only male mice were used in this study [11, 44].

Nevertheless, DOX-induced cognitive impairment also has a high incidence in female patients. As we know, chemotherapy is more toxic in females than in males [45], and the response to immunotherapy is poorer in females [46]. The mechanisms underlying gender differences are multifaceted, encompassing factors that are both dependent on and independent of sex steroid hormones [47]. For instance, compared with males, females show a more robust inflammatory response and heightened T cell responses [48]. Besides, mitochondrial dysfunction may be an important driver of sex differences in neuroprotection [49]. Therefore, more investigations in female animal models are needed. Third, although LY294002 can cross the blood-brain barrier, its systemic administration may affect other organs. Future studies should employ cell-specific knockdowns of pathway components to enhance specificity.

CONCLUSION

Overall, the current study investigated the direct neurotoxic mechanisms of DOX-induced CRCI by integrating network toxicology, transcriptomic analysis, and *in vivo* experiments. Our study demonstrated that DOX exerts cognitive damage via the PI3K-AKT-VEGF signaling pathway, which is closely linked to the disruption of the blood-brain barrier. Furthermore, our study indicated that PI3K-AKT-VEGF pathway inhibitors might become promising candidates for the prevention or treatment of DOX-induced CRCI. Given the essential role of astrocytes in BBB integrity, future work should deeply explore the effects of DOX on astrocytic function, which may reveal novel strategies to counteract CRCI.

AUTHORS' CONTRIBUTIONS

The authors confirm their contributions to the paper as follows: XX and YY conceived and designed the study; FMS and CCZ contributed to the conceptualization; XCZ and LCJ collected the data; and JXS, JCP, and KN drafted the manuscript, with all authors reviewing the results and approving the final version.

LIST OF ABBREVIATIONS

BBB	=	Blood-brain Barrier
CRCI	=	Chemotherapy-related Cognitive Impairment
GO	=	Gene Ontology
KEGG	=	Kyoto Encyclopedia of Genes and Genomes
MWM	=	Morris Water Maze
PPI	=	Protein-protein Interaction
SMILES	=	Simplified Molecular Input Line Entry System
VEGF	=	Vascular Endothelial Growth Factor

ETHICS APPROVAL AND CONSENT TO PARTICIPATE

All experimental studies in mice were reviewed and approved by the Institutional Animal Care and Use Committee of Shanghai Tenth People's Hospital of Tongji University, Shanghai, China (Approval number: SHDSYY-2022-4835).

HUMAN AND ANIMAL RIGHTS

This study adheres to internationally accepted standards for animal research and follows the 3Rs principle. The ARRIVE guidelines were employed for reporting experiments involving live animals, promoting ethical research practices.

The animal experimentation was conducted in accordance with the US National Research Council's "Guide for the Care and Use of Laboratory Animals".

CONSENT FOR PUBLICATION

Not applicable.

AVAILABILITY OF DATA AND MATERIALS

The data that support the findings of this study are available from the corresponding author upon reasonable request.

FUNDING

This work was supported by the grants from the National Natural Science Foundation (Grant no. 82204537, to Can-can Zhou), Fundamental Research Funds for the Central Universities (Grant no. 22120220559, to Can-can Zhou), and Shanghai Science and Technology Innovation Action Plan (Grant no. 24SF1900902, to Fu-Ming Shen).

CONFLICT OF INTEREST

The authors declare no conflict of interest, financial or otherwise.

ACKNOWLEDGEMENTS

Graphical abstract was created by Figdraw (www.figdraw.com).

SUPPLEMENTARY MATERIAL

Supplementary material is available on the publisher's website along with the published article.

REFERENCES

[1] He, Y.Q.; Zhou, C.C.; Jiang, S.G.; Lan, W.Q.; Zhang, F.; Tao, X.; Chen, W.S. Natural products for the treatment of chemotherapy-related cognitive impairment and prospects of nose-to-brain drug delivery. *Front. Pharmacol.,* **2024**, *15*, 1292807. http://dx.doi.org/10.3389/fphar.2024.1292807 PMID: 38348396

[2] Hutchinson, A.D.; Pfeiffer, S.M.; Wilson, C. Cancer-related cognitive impairment in children. *Curr. Opin. Support Palliat. Care,* **2017**, *11*(1), 70-75. http://dx.doi.org/10.1097/SPC.0000000000000258 PMID: 28009650

[3] Khan, M.A.; Garg, K.; Bhurani, D.; Agarwal, N.B. Early manifestation of mild cognitive impairment in B-cell non-Hodgkin's lymphoma patients receiving CHOP and rituximab-CHOP chemother-

apy. *Naunyn Schmiedebergs Arch. Pharmacol.,* **2016**, *389*(12), 1253-1265.
http://dx.doi.org/10.1007/s00210-016-1290-y PMID: 27568029

[4] Juan, Z.; Chen, J.; Ding, B.; Yongping, L.; Liu, K.; Wang, L.; Le, Y.; Liao, Q.; Shi, J.; Huang, J.; Wu, Y.; Ma, D.; Ouyang, W.; Tong, J. Probiotic supplement attenuates chemotherapy-related cognitive impairment in patients with breast cancer: A randomised, double-blind, and placebo-controlled trial. *Eur. J. Cancer,* **2022**, *161*, 10-22.
http://dx.doi.org/10.1016/j.ejca.2021.11.006 PMID: 34896904

[5] Inbaraj, G.; Sathyaprabha, T.N.; Udupa, K.; Ram, A.; Patil, S.; Rajeswaran, J.; Nandakumar, K.K.; Belur, S.; Singh, A.D.; Prathyusha, P.V.; Bayari, S.K.; Raghavendra, R.M. Impact of integrated yoga therapy on cognitive impairment and cardiac dysfunction in relation to quality of life in breast cancer patients undergoing chemotherapy: Study protocol for a two-arm randomized controlled trial. *Front. Oncol.,* **2022**, *12*, 955184.
http://dx.doi.org/10.3389/fonc.2022.955184 PMID: 36185291

[6] Janelsins, M.C.; Kohli, S.; Mohile, S.G.; Usuki, K.; Ahles, T.A.; Morrow, G.R. An update on cancer- and chemotherapy-related cognitive dysfunction: Current status. *Semin Oncol.,* **2011**, *38*(3), 431-438.
http://dx.doi.org/10.1053/j.seminoncol.2011.03.014 PMID: 21600374

[7] Janelsins, M.C.; Van Dyk, K.; Hartman, S.J.; Koll, T.T.; Cramer, C.K.; Lesser, G.J.; Barton, D.L.; Mustian, K.M.; Wagner, L.I.; Ganz, P.A.; Cole, P.D.; Bakos, A.; Root, J.C.; Hardy, K.; Magnuson, A.; Ferguson, R.J.; McDonald, B.C.; Saykin, A.J.; Gonzalez, B.D.; Wefel, J.S.; Morilak, D.A.; Dahiya, S.; Heijnen, C.J.; Conley, Y.P.; Morgans, A.K.; Mabbott, D.; Monje, M.; Rapp, S.R.; Gondi, V.; Bender, C.; Embry, L.; McCaskill Stevens, W.; Hopkins, J.O.; St Germain, D.; Dorsey, S.G. The National Cancer Institute clinical trials planning meeting to address gaps in observational and intervention trials for cancer-related cognitive impairment. *J. Natl Cancer Inst,* **2025**, *117*(2), 217-228.
http://dx.doi.org/10.1093/jnci/djae209 PMID: 39250738

[8] Jang, H.M.; Lee, J.Y.; An, H.S.; Ahn, Y.J.; Jeong, E.A.; Shin, H.J.; Kim, K.E.; Lee, J.; Koh, J.S.; Roh, G.S. LCN2 deficiency ameliorates doxorubicin-induced cardiomyopathy in mice. *Biochem. Biophys. Res. Commun.,* **2022**, *588*, 8-14.
http://dx.doi.org/10.1016/j.bbrc.2021.12.048 PMID: 34933182

[9] Du, J.; Zhang, A.; Li, J.; Liu, X.; Wu, S.; Wang, B.; Wang, Y.; Jia, H. Doxorubicin-induced cognitive impairment: The mechanistic insights. *Front. Oncol.,* **2021**, *11*, 673340.
http://dx.doi.org/10.3389/fonc.2021.673340 PMID: 34055643

[10] Licht, T.; Sasson, E.; Bell, B.; Grunewald, M.; Kumar, S.; Kreisel, T.; Ben-Zvi, A.; Keshet, E. Hippocampal neural stem cells facilitate access from circulation *via* apical cytoplasmic processes. *eLife,* **2020**, *9*, 52134.
http://dx.doi.org/10.7554/eLife.52134 PMID: 32496193

[11] Ren, X.; Keeney, J.T.R.; Miriyala, S.; Noel, T.; Powell, D.K.; Chaiswing, L.; Bondada, S.; St Clair, D.K.; Butterfield, D.A. The triangle of death of neurons: Oxidative damage, mitochondrial dysfunction, and loss of choline-containing biomolecules in brains of mice treated with doxorubicin. Advanced insights into mechanisms of chemotherapy induced cognitive impairment ("chemobrain") involving TNF-α. *Free Radic Biol. Med.,* **2019**, *134*, 1-8.
http://dx.doi.org/10.1016/j.freeradbiomed.2018.12.029 PMID: 30593843

[12] Sardi, I.; la Marca, G.; Cardellicchio, S.; Giunti, L.; Malvagia, S.; Genitori, L.; Massimino, M.; de Martino, M.; Giovannini, M.G. Pharmacological modulation of blood-brain barrier increases permeability of doxorubicin into the rat brain. *Am. J. Cancer Res.,* **2013**, *3*(4), 424-432.
PMID: 23977451

[13] Manchon, J.F.M.; Dabaghian, Y.; Uzor, N.E.; Kesler, S.R.; Wefel, J.S.; Tsvetkov, A.S. Levetiracetam mitigates doxorubicin-induced DNA and synaptic damage in neurons. *Sci. Rep.,* **2016**, *6*(1), 25705.
http://dx.doi.org/10.1038/srep25705 PMID: 27168474

[14] Park, H.S.; Kim, C.J.; Kwak, H.B.; No, M.H.; Heo, J.W.; Kim, T.W. Physical exercise prevents cognitive impairment by enhancing hippocampal neuroplasticity and mitochondrial function in doxorubicin-induced chemobrain. *Neuropharmacology,* **2018**, *133*, 451-461.
http://dx.doi.org/10.1016/j.neuropharm.2018.02.013 PMID: 29477301

[15] Xiao, F.; Qiu, J.; Zhao, Y. Exploring the potential toxicological mechanisms of vine tea on the liver based on network toxicology and transcriptomics. *Front. Pharmacol.,* **2022**, *13*, 855926.
http://dx.doi.org/10.3389/fphar.2022.855926 PMID: 35392562

[16] Yang, M.; Mao, K.; Cao, X.; Liu, H.; Mao, W.; Hao, L. Integrated network toxicology, transcriptomics and gut microbiomics reveals hepatotoxicity mechanism induced by benzo[a]pyrene exposure in mice. *Toxicol. Appl. Pharmacol.,* **2024**, *491*, 117050.
http://dx.doi.org/10.1016/j.taap.2024.117050 PMID: 39111554

[17] Thomas, T.C.; Beitchman, J.A.; Pomerleau, F.; Noel, T.; Jungsuwadee, P.; Butterfield, D.A.; Clair, D.K.S.; Vore, M.; Gerhardt, G.A. Acute treatment with doxorubicin affects glutamate neurotransmission in the mouse frontal cortex and hippocampus. *Brain Res.,* **2017**, *1672*, 10-17.
http://dx.doi.org/10.1016/j.brainres.2017.07.003 PMID: 28705715

[18] Gong, Z.; Lao, D.; Wu, Y.; Li, T.; Lv, S.; Mo, X.; Huang, W. Inhibiting PI3K/Akt-signaling pathway improves neurobehavior changes in Anti-NMDAR encephalitis mice by ameliorating blood–brain barrier disruption and neuronal damage. *Cell. Mol. Neurobiol.,* **2023**, *43*(7), 3623-3637.
http://dx.doi.org/10.1007/s10571-023-01371-3 PMID: 37314618

[19] Wang, Y.; Yuan, Y.; Wang, W.; He, Y.; Zhong, H.; Zhou, X.; Chen, Y.; Cai, X.J.; Liu, L. Mechanisms underlying the therapeutic effects of Qingfeiyin in treating acute lung injury based on GEO datasets, network pharmacology and molecular docking. *Comput. Biol. Med.,* **2022**, *145*, 105454.
http://dx.doi.org/10.1016/j.compbiomed.2022.105454 PMID: 35367781

[20] He, Q.; Liu, C.; Wang, X.; Rong, K.; Zhu, M.; Duan, L.; Zheng, P.; Mi, Y. Exploring the mechanism of curcumin in the treatment of colon cancer based on network pharmacology and molecular docking. *Front. Pharmacol.,* **2023**, *14*, 1102581.
http://dx.doi.org/10.3389/fphar.2023.1102581 PMID: 36874006

[21] Huang, R.; Okyere, S.K.; Shao, C.; Yousif, M.; Liao, F.; Wang, X.; Wen, J.; Wang, J.; Hu, Y. Hepatotoxicity effects of Ageratina adenophora, as indicated by network toxicology combined with metabolomics and transcriptomics. *Ecotoxicol Environ. Saf.,* **2023**, *267*, 115664.
http://dx.doi.org/10.1016/j.ecoenv.2023.115664 PMID: 37948940

[22] Zhou, C.C.; He, Y.Q.; Qiu, Y.S.; Ni, C.X.; Shen, F.M.; Li, D.J. Zinc supplementation ameliorates sorafenib-induced cognitive impairment through ROS/JNK signaling pathway. *Biol. Trace Elem. Res.,* **2023**, *201*(1), 324-337.
http://dx.doi.org/10.1007/s12011-022-03142-5 PMID: 35129807

[23] Guo, Q.; Li, T.F.; Huang, J.; Li, J.C.; Zhang, Z.C.; Qu, Y.L. The protective role of phlorizin against lipopolysaccharide-induced acute orchitis in mice associated with changes in gut microbiota composition. *Front. Vet. Sci.,* **2024**, *11*, 1340591.
http://dx.doi.org/10.3389/fvets.2024.1340591 PMID: 38846786

[24] Yang, K.; Wu, J.; Li, S.; Wang, S.; Zhang, J.; Wang, Y.; Yan, Y.; Hu, H.; Xiong, M.; Bai, C.; Sun, Y.; Chen, W.; Zeng, Y.; Yuan, J.; Yin, C. NTRK1 knockdown induces mouse cognitive impairment and hippocampal neuronal damage through mitophagy suppression *via* inactivating the AMPK/ULK1/FUNDC1 pathway. *Cell. Death Discov.,* **2023**, *9*(1), 404.
http://dx.doi.org/10.1038/s41420-023-01685-7 PMID: 37907480

[25] Jinpiao, Z.; Zongze, Z.; Qiuyue, Y.; Peng, F.; Qi, Z.; Yanlin, W.; Chang, C. Metformin attenuates sevoflurane-induced neurocognitive impairment through AMPK-ULK1-dependent autophagy in aged mice. *Brain Res. Bull,* **2020**, *157*, 18-25.
http://dx.doi.org/10.1016/j.brainresbull.2020.01.018 PMID: 32014566

[26] Fei, Y.; Ling, Z.; Tong, Q.; Wang, J. Apoptotic extracellular vesicles from supernumerary tooth-derived pulp stem cells transfer col1a1 to promote angiogenesis *via* PI3K/Akt/VEGF pathway. *Int. J. Nanomed.,* **2024**, *19*, 6811-6828.
http://dx.doi.org/10.2147/IJN.S466136 PMID: 39005959

[27] Prathumsap, N.; Shinlapawittayatorn, K.; Chattipakorn, S.C.; Chattipakorn, N. Effects of doxorubicin on the heart: From molecular

mechanisms to intervention strategies. *Eur. J. Pharmacol.,* **2020**, *866*, 172818.
http://dx.doi.org/10.1016/j.ejphar.2019.172818 PMID: 31758940

[28] Navarro-Hortal, M.D.; Varela-López, A.; Romero-Márquez, J.M.; Rivas-García, L.; Speranza, L.; Battino, M.; Quiles, J.L. Role of flavonoids against adriamycin toxicity. *Food Chem. Toxicol.,* **2020**, *146*, 111820.
http://dx.doi.org/10.1016/j.fct.2020.111820 PMID: 33080329

[29] Rawat, P.S.; Jaiswal, A.; Khurana, A.; Bhatti, J.S.; Navik, U. Doxorubicin-induced cardiotoxicity: An update on the molecular mechanism and novel therapeutic strategies for effective management. *Biomed. Pharmacother.,* **2021**, *139*, 111708.
http://dx.doi.org/10.1016/j.biopha.2021.111708 PMID: 34243633

[30] Huang, S. A novel strategy for the study on molecular mechanism of prostate injury induced by 4,4′-sulfonyldiphenol based on network toxicology analysis. *J. Appl. Toxicol.,* **2024**, *44*(1), 28-40.
http://dx.doi.org/10.1002/jat.4506 PMID: 37340727

[31] Ge, B.; Yan, K.; Sang, R.; Wang, W.; Liu, X.; Yu, M.; Liu, X.; Qiu, Q.; Zhang, X. Integrated network toxicology, molecular docking, and *in vivo* experiments to elucidate molecular mechanism of aflatoxin B1 hepatotoxicity. *Ecotoxicol. Environ. Saf.,* **2024**, *275*, 116278.
http://dx.doi.org/10.1016/j.ecoenv.2024.116278 PMID: 38564860

[32] Rufini, A.; Tucci, P.; Celardo, I.; Melino, G. Senescence and aging: The critical roles of p53. *Oncogene,* **2013**, *32*(43), 5129-5143.
http://dx.doi.org/10.1038/onc.2012.640 PMID: 23416979

[33] Qian, X.; Liu, X.; Chen, S.; Tang, H. Integrating peripheral blood and brain transcriptomics to identify immunological features associated with Alzheimer's disease in mild cognitive impairment patients. *Front. Immunol.,* **2022**, *13*, 986346.
http://dx.doi.org/10.3389/fimmu.2022.986346 PMID: 36159817

[34] Zhang, S.; Li, M.; Qiu, Y.; Wu, J.; Xu, X.; Ma, Q.; Zheng, Z.; Lu, G.; Deng, Z.; Huang, H. Enhanced VEGF secretion and blood–brain barrier disruption: Radiation-mediated inhibition of astrocyte autophagy *via* PI3K-AKT pathway activation. *Glia,* **2024**, *72*(3), 568-587.
http://dx.doi.org/10.1002/glia.24491 PMID: 38009296

[35] Matsuno, H.; Tsuchimine, S.; O'Hashi, K.; Sakai, K.; Hattori, K.; Hidese, S.; Nakajima, S.; Chiba, S.; Yoshimura, A.; Fukuzato, N.; Kando, M.; Tatsumi, M.; Ogawa, S.; Ichinohe, N.; Kunugi, H.; Sohya, K. Association between vascular endothelial growth factor-mediated blood–brain barrier dysfunction and stress-induced depression. *Mol. Psychiatry,* **2022**, *27*(9), 3822-3832.
http://dx.doi.org/10.1038/s41380-022-01618-3 PMID: 35618888

[36] Argaw, A.T.; Asp, L.; Zhang, J.; Navrazhina, K.; Pham, T.; Mariani, J.N.; Mahase, S.; Dutta, D.J.; Seto, J.; Kramer, E.G.; Ferrara, N.; Sofroniew, M.V.; John, G.R. Astrocyte-derived VEGF-A drives blood-brain barrier disruption in CNS inflammatory disease. *J. Clin. Invest.,* **2012**, *122*(7), 2454-2468.
http://dx.doi.org/10.1172/JCI60842 PMID: 22653056

[37] Hu, D.; Mo, X.; Luo, J.; Wang, F.; Huang, C.; Xie, H.; Jin, L. 17-DMAG ameliorates neuroinflammation and BBB disruption *via* SOX5 mediated PI3K/Akt pathway after intracerebral hemorrhage in rats. *Int. Immunopharmacol.,* **2023**, *123*, 110698.
http://dx.doi.org/10.1016/j.intimp.2023.110698 PMID: 37517381

[38] Zhu, Q.; Enkhjargal, B.; Huang, L.; Zhang, T.; Sun, C.; Xie, Z.; Wu, P.; Mo, J.; Tang, J.; Xie, Z.; Zhang, J.H. Aggf1 attenuates neuroinflammation and BBB disruption *via* PI3K/Akt/NF-κB pathway after subarachnoid hemorrhage in rats. *J. Neuroinflammation,* **2018**, *15*(1), 178.
http://dx.doi.org/10.1186/s12974-018-1211-8 PMID: 29885663

[39] Gao, Q.; Hernandes, M.S. Sepsis-associated encephalopathy and blood-brain barrier dysfunction. *Inflammation,* **2021**, *44*(6), 2143-2150.
http://dx.doi.org/10.1007/s10753-021-01501-3 PMID: 34291398

[40] Hu, Y.; Hu, X.; He, Z.; Liu, Y.; Gui, Y.; Zhu, S.; Da, X.; Liu, Y.; Liu, L.; Shen, Q.; Xu, G. Anesthesia/surgery activate MMP9 leading to blood-brain barrier disruption, triggering neuroinflammation and POD-like behavior in aged mice. *Int. Immunopharmacol.,* **2024**, *135*, 112290.
http://dx.doi.org/10.1016/j.intimp.2024.112290 PMID: 38796964

[41] Wang, F.; Li, R.; Tu, P.; Chen, J.; Zeng, K.; Jiang, Y. Total glycosides of *Cistanche deserticola* promote neurological function recovery by inducing neurovascular regeneration *via* Nrf-2/Keap-1 pathway in MCAO/R rats. *Front. Pharmacol.,* **2020**, *11*, 236.
http://dx.doi.org/10.3389/fphar.2020.00236 PMID: 32256351

[42] Garabadu, D.; Verma, J. Exendin-4 attenuates brain mitochondrial toxicity through PI3K/Akt-dependent pathway in amyloid beta (1-42)-induced cognitive deficit rats. *Neurochem. Int.,* **2019**, *128*, 39-49.
http://dx.doi.org/10.1016/j.neuint.2019.04.006 PMID: 31004737

[43] Lefrak, E.A.; Pit'ha, J.; Rosenheim, S.; Gottlieb, J.A. A clinicopathologic analysis of adriamycin cardiotoxicity. *Cancer,* **1973**, *32*(2), 302-314.
http://dx.doi.org/10.1002/1097-0142(197308)32:2<302::AID-CNCR2820320205>3.0.CO;2-2 PMID: 4353012

[44] Wang, C.; Zhao, Y.; Wang, L.; Pan, S.; Liu, Y.; Li, S.; Wang, D. C-phycocyanin mitigates cognitive impairment in doxorubicin-induced chemobrain: Impact on neuroinflammation, oxidative stress, and brain mitochondrial and synaptic alterations. *Neurochem. Res.,* **2021**, *46*(2), 149-158.
http://dx.doi.org/10.1007/s11064-020-03164-2 PMID: 33237471

[45] Lopes-Ramos, C.M.; Quackenbush, J.; DeMeo, D.L. Genome-wide sex and gender differences in cancer. *Front. Oncol.,* **2020**, *10*, 597788.
http://dx.doi.org/10.3389/fonc.2020.597788 PMID: 33330090

[46] Irelli, A.; Sirufo, M.M.; D'Ugo, C.; Ginaldi, L.; De Martinis, M. Sex and gender influences on cancer immunotherapy response. *Biomedicines,* **2020**, *8*(7), 232.
http://dx.doi.org/10.3390/biomedicines8070232 PMID: 32708265

[47] Rubin, J.B.; Lagas, J.S.; Broestl, L.; Sponagel, J.; Rockwell, N.; Rhee, G.; Rosen, S.F.; Chen, S.; Klein, R.S.; Imoukhuede, P.; Luo, J. Sex differences in cancer mechanisms. *Biol. Sex Differ.,* **2020**, *11*(1), 17.
http://dx.doi.org/10.1186/s13293-020-00291-x PMID: 32295632

[48] Klein, S.L.; Flanagan, K.L. Sex differences in immune responses. *Nat. Rev. Immunol.,* **2016**, *16*(10), 626-638.
http://dx.doi.org/10.1038/nri.2016.90 PMID: 27546235

[49] Demarest, T.G.; McCarthy, M.M. Sex differences in mitochondrial (dys)function: Implications for neuroprotection. *J. Bioenerg. Biomembr.,* **2015**, *47*(1-2), 173-188.
http://dx.doi.org/10.1007/s10863-014-9583-7 PMID: 25293493
